# Protection of Ducklings from Duck Hepatitis A Virus Infection with ELPylated Duck Interferon-α

**DOI:** 10.3390/v14030633

**Published:** 2022-03-18

**Authors:** Yongjuan Wang, Yanli Guo, Haowei Wang, Zhi Wu, Weiming Hong, Huaichang Sun, Shanyuan Zhu

**Affiliations:** 1 Department of Veterinary Medicine, Jiangsu Agri-Animal Husbandry Vocational College, Taizhou 225300, China; wangyj2022022@163.com (Y.W.); yl425244808@163.com (Y.G.); 13160266269@139.com (H.W.); yzwuzhi@163.com (Z.W.); jsahvc@163.com (W.H.); 2 College of Veterinary Medicine, Jiangsu Co-Innovation Center for Prevention and Control of Important Animal Infectious Diseases and Zoonoses, Yangzhou University, Yangzhou 225009, China

**Keywords:** duck interferon-α, elastin-like polypeptide, fusion expression, interferon-stimulated gene, duck viral hepatitis, antiviral reagent

## Abstract

Duck viral hepatitis type I (DVH I) is a lethal disease in ducklings caused by duck hepatitis A virus (DHAV). Although the commercial vaccine is available for vaccination of one-day-old ducklings or breeder ducks, the disease is still prevalent due to the delayed immune response in ducklings and variable maternal antibody levels in breeder duck flocks. To explore the feasibility of duck interferon-α (DuIFN-α) for control of DVH I, DuIFN-α was expressed as an elastin-like polypeptide (ELP) fusion protein (ELP-DuIFN-α) in *E. coli* and purified by inverse phase transition cycling (ITC). After detection of its cytotoxicity, bioactivity, plasma stability and serum half-life, the protective efficacy of ELP-DuIFN-α against DHAV-1 infection of embryos or ducklings was evaluated using different treatment routes at different infection times. The results show that ELP-DuIFN-α was correctly expressed and purified to more than 90% purity after two cycles of ITC. The purified fusion protein had a specific anti-DHAV-1 activity of 6.0 × 10^4^ IU/mg protein, significantly extended plasma stability and serum half-life without overt cytotoxicity. After allantoic injection with ELP-DuIFN-α pre-infection, co-infection or post-infection with DHAV-1, 5/5, 5/5 or 4/5 embryos survived from the virus challenge. After intramuscular injection or oral administration with ELP-DuIFN-α, 3/5 or 4/5 ducklings survived from co-infection with DHAV-1. After oral administration with ELP-DuIFN-α pre-infection, co-infection or post-infection with DHAV-1, 3/5, 4/5 or 4/5 ducklings survived from the virus challenge, and the relative transcription levels of interferon-stimulated genes were significantly higher than the normal control group and virus challenge control group (*p* < 0.01). These experimental data suggest that ELP-DuIFN-α can be used as a long-lasting anti-DHAV-1 reagent.

## 1. Introduction

Duck viral hepatitis type I (DVH I) is a lethal disease in ducklings that is characterized by liver enlargement, necrosis and hemorrhage [1,2]. The causative agent, duck hepatitis A virus (DHAV), can be classified into three serotypes: DHAV-1, DHAV-2 and DHAV-3. DHAV-1 is the most prevalent serotype, causing major economic loss to the duck industry in China [3,4,5]. Although the disease can be controlled by vaccination of one-day-old ducklings or breeder ducks, the immune response in ducklings is not induced until 3–5 days after vaccination, and the ducklings during the interim face a great risk of the viral infection [6]. Although vaccination of breeder ducks can protect ducklings from DHAV infection during the interim, maintaining proper maternal antibody levels in large flocks can be difficult. Therefore, there is an urgent need to develop a new strategy against DHAV infection [7].

Interferons (IFNs) are a class of cytokines with strong antiviral and immune regulatory activities [8,9]. Among the three types of IFNs, IFN-α is the prototype of type I IFNs with broad antiviral activities [10,11]. Since its first gene cloning, recombinant duck IFN-α (rDuIFN-α) has been expressed in mammalian cells and used as the antiviral reagent against avian influenza virus and duck hepatitis B virus [12,13,14]. However, the expression of rDuIFN-α in mammalian cells requires time-consuming drug selection for stably transfected cells. In addition, the clinical use of such rDuIFN-α is limited by low expression level, difficult purification and short serum half-life [15,16].

Elastin-like polypeptides (ELPs) are genetically synthetic biopolymers composed of Val-Pro-Gly-Xaa-Gly repeats, where the guest residue Xaa can be any amino acid except proline [17]. These synthetic polymers can undergo reversible phase transition from soluble monomers into protein aggregates as temperature increases [18]. This unique property, together with the excellent biocompatibility and low immunogenicity, makes ELPs very useful for a wide variety of biomedical applications, including protein purification and drug delivery [19]. More recently, ELP fusion technology has been used to enhance the pharmacokinetics and bioactivity of human IFN-α and pig IFN-α [20,21]. Therefore, the main objective of this study was to explore the feasibility of ELP-DuIFN-α for control of DVH I.

## 2. Materials and Methods

### 2.1. Protein Expression

ELP fusion expression vector pET-ELP was constructed by cloning the coding sequence for 110 repeats of VPGVG block into pET-30a (+) vector with *Nde*I and *Sac*I digestion [22]. The coding sequence for the mature peptide of DuIFN-α (GenBank accession: DQ861429) was adapted to *E. coli* codon usage using the Java Codon Adaption Tool [23]. The synthetic sequence was cloned into the pET-ELP vector with *Sac*I and *Xho*I digestion. The recombinant pELP-DuIFNα vector was transformed into *E. coli* strain BL21 (DE3), and Luria broth culture containing kanamycin (50 μg/mL) was grown overnight at 37 °C. The bacterial culture was diluted (1:100) in 2 × YT medium (10 g yeast extract, 16 g tryptone, 5 g NaCl/L, pH 7.2) containing the same antibiotic. After growth for 5 h at 37 °C, the expression of ELP-DuIFN-α fusion protein was induced with 0.2 mM IPTG for 8 h at 20 °C.

### 2.2. Protein Purification

The inverse transition cycling (ITC) for ELP-DuIFN-α purification was performed as previously described [21]. Briefly, IPTG-induced *E. coli* cells were harvested by centrifugation, washed one time and suspended in PBS (pH 7.2). After sonication treatment, the cell lysate was centrifuged for 20 min at 12,000× *g*, and the supernatant was collected for protein purification. To determine the transition temperature of ELP-DuIFN-α fusion protein, the cell lysate was incubated with an equal volume of 6M NaCl for 10 min at different temperatures (26 °C, 28 °C, 30 °C). To optimize the salt concentration for ITC, the cell lysate was incubated with different concentrations(1M, 2M, 3M) of NaCl for 10 min at the optimized temperature. After 5 min centrifugation at 12,000× *g* at room temperature (hot spin), the protein pellet was suspended in cold PBS and incubated on ice until complete dissolution. After additional 10 min centrifugation at 12,000× *g* at 4 °C (cold spin), the soluble protein was collected. The protein samples before and after ITC were analyzed by 12% SDS-PAGE for protein recovery and purity using Molecular Imager^®^ Gel Doc™ XR+ System with Image Lab™ Software (BIO-RAD). Finally, ELP-DuIFN-α was purified from 1 L of bacterial culture under the optimized conditions.

### 2.3. Cytotoxicity Assay

The cytotoxicity of ELP-DuIFN-α was detected using a MTT Cell Proliferation and Cytotoxicity Assay Kit (CWBIO, Beijing, China). Briefly, MDCK cell line (ATCC CCL-34) or duck embryonic fibroblast (DEF) cells were seeded on 96-well plates and grown to 90% confluence in DMEM supplemented with 10% fetal bovine serum (FBS). ELP-DuIFN-α (2 mg/mL) was serially diluted with the cell medium and added to the wells (100 µL/well). After 72 h incubation at 37 °C, MTT agent was added, and OD_490_ values were measured on an ELISA microplate reader. The cell growth inhibition rates of ELP-DuIFN-α were calculated according to the formula: growth inhibition rate (%) = (OD_490_ value of mock-treated cells−OD_490_ value of ELP-DuIFN-α-treated cells)/OD_490_ value of mock-treated cells × 100% (n = 3).

### 2.4. Virus Proliferation

Virus DHAV-1-SH (supplied by the Shanghai Veterinary Research Institute of the Chinese Academy of Agricultural Sciences) was proliferated on DEF cells. Briefly, DEF cells were seeded on a cell plate and infected with DHAV-1 when grown to 90% confluence in DMEM supplemented with 10% FBS. Three days after DHAV-1 infection, both the superment and cells were collected for DHAV-1 harvesting. Then 50% tissue culture infection dose (TCID_50_), 50% egg lethal dose (ELD_50_) and 50% lethal dose (LD_50_) of DHAV-1 were respectively calculated by Karber’s method on DEF cells, embryos or ducklings. At the same time, proliferation, harvest and quantitation of VSV were carried on MDCK cells.

### 2.5. In Vitro Antiviral Assay

The in vitro antiviral activities of ELP-DuIFN-α against vesicular stomatitis virus (VSV) and DHAV-1 were measured by cytopathic effect (CPE) inhibition assay as previously described [24]. Briefly, the cells were seeded on 96-well plates (8 × 10^3^ cells/well) and grown to 90% confluence. ELP-DuIFN-α (2 mg/mL) was serially diluted in PBS (pH 7.2) and added to the wells (100 μL/well) in six duplicates. After 24 h incubation at 37 °C, the optimal dose (100 TCID_50_) of virus was added and incubated for additional 24 h. The antiviral activity of ELP-DuIFN-α was calculated according to the Read–Muench algorithm and expressed as IU/mg protein. To confirm the antiviral activity, DEF cells were mock-treated or treated with ELP-DuIFN-α (6 × 10^4^ IU/well) and then infected with VSV or DHAV-1 as described. At 24 h post infection (hpi), the CPE was observed under light microscope.

### 2.6. Plasma Stability Assay

The plasma stability of ELP-DuIFN-α was measured by CPE inhibition assay as described [24]. Briefly, the purified protein was diluted in 50% duck plasma (10 μg/mL) and incubated for 0.25, 0.5, 1, 3, 6, 12, 24 or 48 h at 37 °C (n = 3). After 20 min centrifugation at 12,000× *g*, the supernatant was collected, and the remaining anti-VSV activity was assayed on MDCK cells as described.

### 2.7. Serum Half-Life Assay

Ten 4-day-old Cherry Valley ducklings were randomly assigned into two groups (five in each group). The ducklings in the control group were orally administered with 0.2 mL of PBS. The ducklings in the experimental group were orally administered with 0.2 mL (1920 IU) of ELP-DuIFN-α. The serum samples were collected at 0, 6, 12, 24, 36, 48, 72 or 96 h, and the remaining anti-VSV activity was assayed on MDCK cells as described (n = 3).

### 2.8. Virus Infection Protection Assay in Embryos

The protective effect of ELP-DuIFN-α against DHAV-1 infection of embryos was detected as previously described [25]. Briefly, twenty-five 9-day-old duck embryos were randomly divided into 5 groups (five in each group). The normal control embryos were allantoically injected with 100 μL of PBS. The virus challenge control embryos were injected with 100 μL (100 ELD_50_) of DHAV-1. The embryos in treatments 1–3 were injected with 100 μL (200 μg) of ELP-DuIFN-α pre-infection (24 h), co-infection or post-infection (24 h) with DHAV-1 (100 ELD_50_). All embryos were observed daily for embryo death. At day 7 post infection, the allantoic fluids were collected from live embryos and passed for 3 generations in SPF chicken embryos. Total RNA was extracted from the allantoic fluids of dead duck embryos and virally passaged chicken embryos using Viral RNA/DNA Extraction Kit (TaKaRa). PCR was performed using DHAV-1 *vp1*-specific forward primer (5′-GTTTGGGAGGCAATGGTT-3′) and reverse primer (5′-ATTGAGTCCACATGAACAG-3′) as previously described [26].

### 2.9. Virus Infection Protection Assay in Ducklings

In experiment 1, twenty 4-day-old Cherry Valley ducklings were divided into four groups (five in each group). The normal control ducklings were orally administered with 200 μL of PBS. The virus challenge control ducklings were orally inoculated with 200 μL (100 LD_50_) of DHAV-1. The experimental ducklings were intramuscularly injected (treatment 1) or orally administered (treatment 2) with ELP-DuIFN-α (160 μg/kg) and DHAV-1 (100 LD_50_) at the same time (co-infection). In experiment 2, fifteen ducklings were assigned into three groups (five in each group) and orally administered with the same dose of ELP-DuIFN-α pre-infection (24 h, treatment 3), co-infection (treatment 4) or post-infection (24 h, treatment 5) with DHAV-1 (100 LD_50_). The same dose of ELP-DuIFN-α was orally administered in two-day intervals for two more times. All ducklings were observed daily for DVH I symptoms, and the dead ducklings were observed for gross lesions.

### 2.10. Interferon Stimulated Genes Assay in Experimental Livers

The livers of ducklings in normal control groups, virus challenge control groups and treatment 3 to 5 groups were collected 3 days after DHAV-1 infection for interferon stimulated gene (ISG) assay. Total RNA was extracted from the livers using a Viral RNA/DNA Extraction Kit (TaKaRa). Relative quantitative PCR for glyceraldehyde-3-phosphate dehydrogenase (GAPDH), 2’-5’oligoadenylate synthetase (OAS), myxovirus resistance protein (Mx), protein kinase R (PKR), zinc-finger antiviral protein (ZAP), interferon-stimulated gene 15 (ISG15) was performed using primers as shown in Table 1.

## 3. Results

### 3.1. ELP-DuIFN-α Expression and Purification

**Figure 1:**
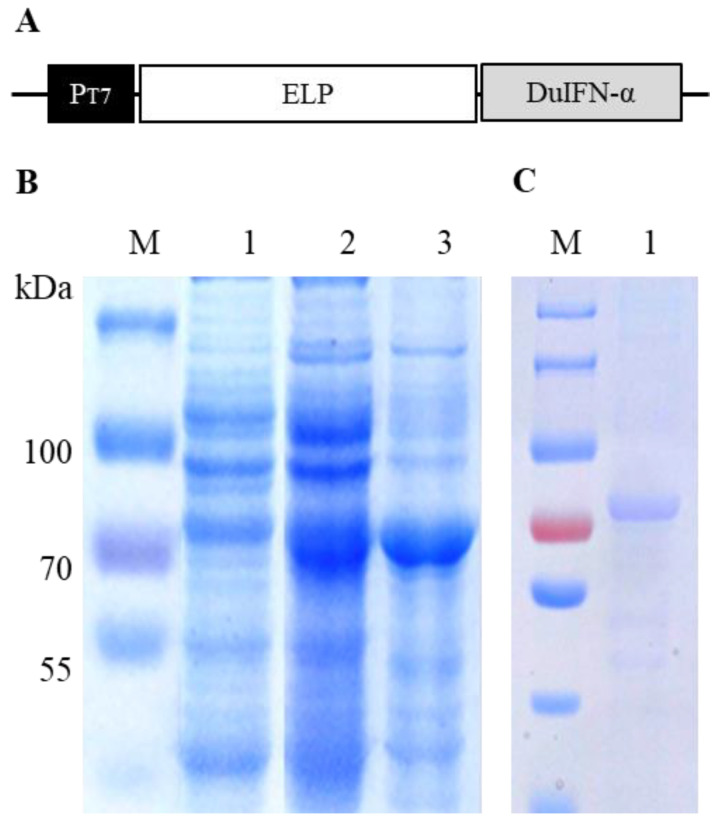
Expression and purification of ELP-DuIFN-α in *E. coli.* (**A**) Schematic structure of pELP-DuIFNα vector. T7 promoter (P_T7_) and the coding sequences for ELP and DuIFN-α are indicated. (**B**) SDS-PAGE analysis of ELP-DuIFN-α expression. M, Protein molecular weight marker; 1, IPTG-induced recombinant *E. coli*; 2, Soluble fraction of centrifuged *E. coli* lysate; 3, Insoluble fraction of centrifuged *E. coli* lysate. (**C**) SDS-PAGE analysis of ELP-DuIFN-α purification. M, Protein molecular weight marker; 1, Purified ELP-DuIFN-α.

Sequencing analysis showed that pELP-DuIFN-α vector (Figure 1A) was correctly constructed without mutation and deletion/insertion. After transformation into *E. coli* strain BL21 (DE3), the expression of ELP-DuIFN-α fusion protein was induced slowly with 0.2 mM IPTG at 20 °C. SDS-PAGE analysis showed that an expected 80 kDa ELP-DuIFN-α fusion protein was expressed in the recombinant vector-transformed *E. coli* but not in the control vector-transformed *E. coli*. More than 60% of the expressed protein was present in the soluble fraction of centrifuged cell lysate (Figure 1B). The optimal transition temperature of ELP-DuIFN-α was 28 °C in 3 M NaCl. After two cycles of ITC, ELP-DuIFN-α was purified to more than 90% purity, with 96% recovery (Figure 1C).

### 3.2. Cytotoxicity of ELP-DuIFN-α

After treatment of MDCK or DEF cells for 72 h with different concentrations of the purified protein, MTT assay showed that ELP-DuIFN-α at a dose up to 200 μg/mL had no overt inhibitory effect on the cell growth (Table 2).

### 3.3. In Vitro Antiviral Activities of ELP-DuIFN-α

**Figure 2:**
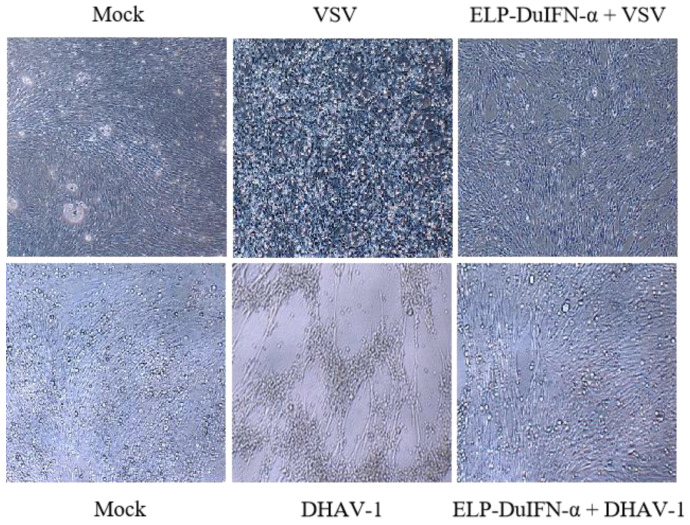
Protection of DEF cells from virus infection by ELP-DuIFN-α. After treatment with ELP-DuIFN-α for 24 h, DEF cells were mock-infected or infected with VSV or DHAV-1 (100 TCID_50_), and observed under light microscope (×400) 24 h post infection.

ELP-DuIFN-α had the specific anti-VSV activity of 1.25 × 10^6^ IU/mg on MDCK cells or 1.25 × 10^7^ IU/mg on DEF cells. On DEF cells, ELP-DuIFN-α had the specific anti-DHAV-1 activity of 6.0 × 10^4^ IU/mg (Table 3). The two virally infected control cells showed severe CPEs, including cell rounding, death and/or detachment. In contrast, no CPE was observed in ELP-DuIFN-α-treated cells at 24 h after infection with VSV or DHAV-1 (Figure 2).

### 3.4. Plasma and Serum Half-Lives of ELP-DuIFN-α

**Figure 3:**
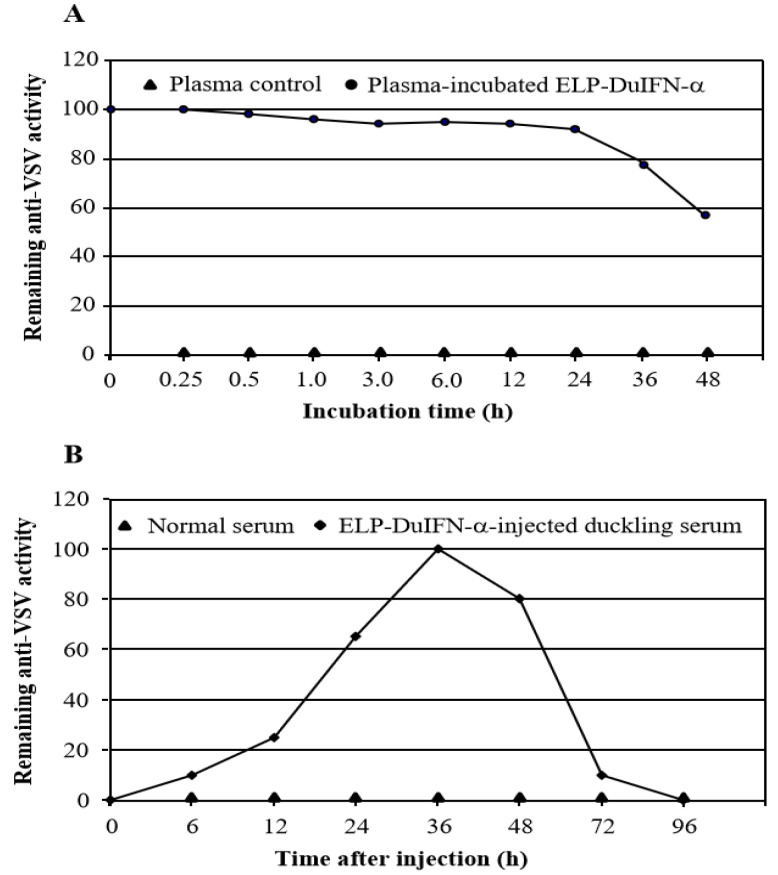
Detection of half-lives of ELP-DuIFN-α. (**A**) ELP-DuIFN-α was incubated in 50% duck plasma for indicated times before anti-VSV activity detection. (**B**) Ducklings were orally administered with ELP-DuIFN-α, and serum samples were collected at the indicated times before anti-VSV activity detection. The remaining antiviral activity was measured by CPE inhibition assay.

After incubation in 50% duck plasma, the remaining anti-VSV activity of ELP-DuIFN-α decreased slowly with the extension in incubation time, with 50% remaining specific antiviral activity at 48 h after incubation (Figure 3A). After oral administration with ELP-DuIFN-α, the anti-VSV activity in duckling serum increased gradually and reached to the highest level by 36 h, with 50% remaining antiviral activity by 60 h after administration (Figure 3B).

### 3.5. Protection of Embryos from DHAV-1 Infection with ELP-DuIFN-α

**Figure 4:**
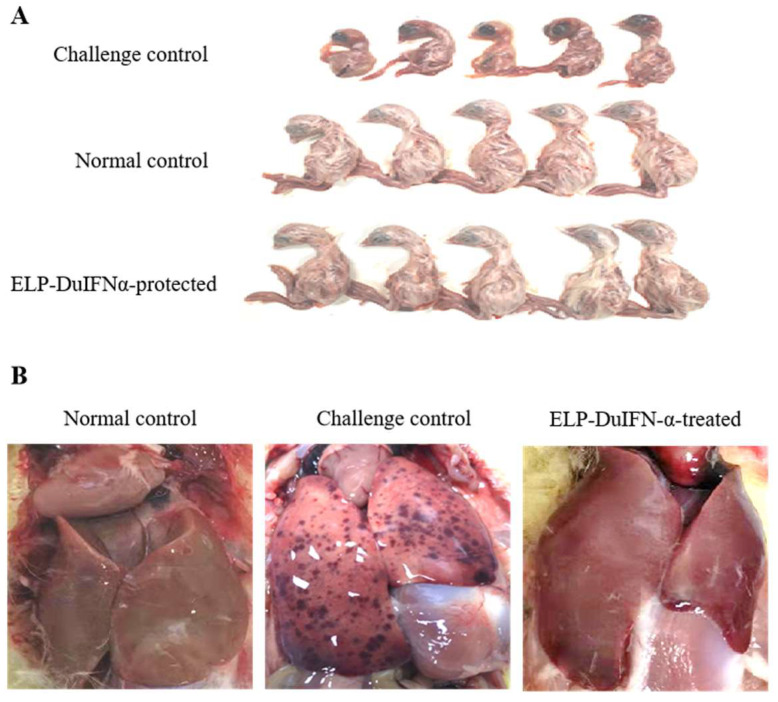
Protection of duck embryos from DHAV-1 infection with ELP-DuIFN-α. (**A**) The five ducklings hatched from normal control embryos, DHAV-1 challenge control embryos or ELP-DuIFNα-treated embryos are shown. (**B**) Comparison of histopathological changes between ducklings hatched from control embryos, challenge control embryos and ELP-DuIFNα-treated embryos.

The five normal control embryos remained healthy until the end of experiment, with five normal ducklings hatched (Figure 4A). All of the five virus challenge control embryos died by day 2 after challenge with DHAV-1 with dead dwarf ducklings (Figure 4A). The dead embryos showed typical DVH I symptoms, including liver enlargement and hemorrhage (Figure 4B). After allantoic injection with ELP-DuIFN-α pre-infection (treatment 1) or co-infection (treatment 2) with DHAV-1, all of the ten embryos survived the virus challenge by day 12, with ten healthy ducklings hatched (Figure 4A). After infection with DHAV-1 (treatment 3), 4/5 embryos were protected from the virus challenge by ELP-DuIFN-α treatment (Figure 4). The protective efficacy of ELP-DuIFN-α against DHAV-1 infection is summarized in Table 4. A specific 360 *bp* *vp1* gene segment was amplified from the embryo that died of the DHAV-1 challenge. In contrast, no specific gene segment was amplified from all of the survived embryos after passaging for three generations in chicken embryos.

Duck embryos in treatments 1–3 were allantoically injected with ELP-DuIFN-α pre-infection, co-infection or post-infection with DHAV-1.

### 3.6. Protection of Ducklings from DHAV-1 Infection with ELP-DuIFN-α

The five normal control ducklings remained healthy at the end of experiment. The five virus challenge control ducklings died by day 3 after DHAV-1 challenge. After intramuscular injection (treatment 1) or oral administration (treatment 2) with ELP-DuIFN-α, 3/5 or 4/5 ducklings survived from co-infection with DHAV-1. After oral administration with ELP-DuIFN-α pre-infection (treatment 3), co-infection (treatment 4) or post-infection (treatment 5) with DHAV-1, 3/5, 4/5 or 4/5 ducklings survived from the virus challenge (Table 5). The dead ducklings showed typical DVH symptoms and gross lesions, including diarrhea, convulsions and liver bleeding. The surviving ducklings remained healthy by the end of experiment without overt DVH symptoms and gross lesions.

Each duckling in challenge control and treatment groups was challenged with 100 LD_50_ of DHAV-1;

Each duckling in treatment groups was administered with ELP-DuIFNα at 160 μg/kg in 2-day intervals for three different times.

### 3.7. ISGs Transcription Level Changes in Livers with ELP-DuIFN-α

**Figure 5:**
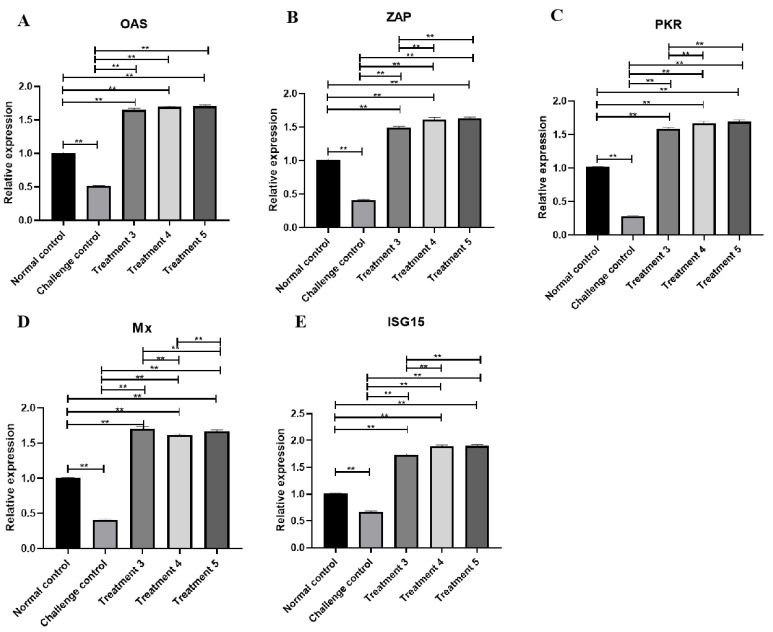
Transcription level of ISGs in livers. (**A**) Relative expression of OAS. (**B**) Relative expression of Mx. (**C**) Relative expression of PKR. (**D**) Relative expression of ZAP. (**E**) Relative expression of ISG15. ** indicates *p* < 0.01.

Taking GAPDH as the internal reference, the transcription levels of ISGs OAS (Figure 5A), Mx (Figure 5B), PKR (Figure 5C), ZAP (Figure 5D) and ISG15 (Figure 5E) in the liver of normal control groups, virus challenge control groups and treatments 3 to 5 groups were measured by RT-PCR, as shown in the Figure 5. Compared with the normal control group and virus challenge control group, the relative transcription levels of treatments 3 to 5 ISGs were significantly higher (*p* < 0.01), and the relative transcription levels of PKR, ZAP and ISG15 in treatments 4 and 5 were higher than that in treatment 3 (*p* < 0.01), while the transcription levels of Mx in treatment 3 were higher than that in treatments 4 and 5 (*p* < 0.01).

## 4. Discussion

Recombinant IFNs have been widely used as the antiviral reagents against a variety of virus infections. Such recombinant IFNs are commonly expressed as affinity-tagged proteins, which require expensive affinity chromatographic columns for purification. In addition, the clinical use of such IFNs is limited by short serum half-lives [16,27]. To simplify the purification of rDuIFN-α and to extend its serum half-life, in this study we expressed DuIFN-α as an ELP fusion protein. After 2 cycles of ITC, ELP-DuIFN-α was purified to more than 90% purity, which could be accomplished within 2 h without the need of expensive equipment and chemicals. Additionally, the purified protein of up to 200 μg/mL had no overt cytotoxicity. Furthermore, it could protect embryos and ducklings from DHAV-1 challenge. These data confirm that ELPs are cost-effective tags for recombinant protein purification [19].

The clinical efficacy of unmodified IFNs may be limited due to their small molecular sizes and rapid clearance from circulation [16,27]. The current strategies for extending the half-lives of small proteins or peptides include polyethylene glycol modification and fusion with large biomolecules such as human serum albumin (HSA). However, the pegylated IFNs have drawbacks of poor efficacy and significant adverse effects [28], while HSA fusions require eukaryotic cells for expression and affinity chromatography for purification [29]. More recently, ELPs have been used to extend serum half-lives of human IFN-α [20] and pig IFN-α [21]. In this study, our plasma stability assay showed that ELP-DuIFN-α was much more stable than unmodified IFN-α. Since IFN-α given orally has biological activity in humans and other animals [30], we measured the serum half-life of orally administered ELP-DuIFN-α in ducklings. As expected, ELP-DuIFN-α had 50% remaining antiviral activity at 60 h after oral administration, which was significantly longer than that of unmodified IFN-α. After oral administration, the efficient absorption of ELP-DuIFN-α into the blood circulation was confirmed by the gradual increase in specific antiviral activity in the duckling serum.

The current tool for DVH I control is to vaccinate one-day-old ducklings or breeder ducks. However, the immune response in ducklings is not induced until 3–5 days after vaccination, and maintaining the proper maternal antibody levels in large flocks is difficult. To explore the feasibility of ELP-DuIFN-α for DVH I control, we first evaluated the protective efficacy in duck embryos since DHAV-1 can be transmitted via the vertical route [31]. After a single dose of injection with ELP-DuIFN-α pre-infection or co-infection with DHAV-1, all embryos survived from the virus challenge, indicating the usefulness of ELP-DuIFN-α for preventing vertical transmission of DHAV-1. Next, we evaluated the protective efficacy of ELP-DuIFN-α against DHAV-1 infection of ducklings using two different administration routes. The results show that oral administration with ELP-DuIFN-α could provide better protection than by intramuscular injection. This was consistent with the previous finding that orally administered IFN-α therapy is a low-dose treatment as compared with the conventional parenteral therapy [30]. Finally, we evaluated the protective efficacy of orally administered ELP-DuIFN-α against DHAV-1 infection of ducklings at different infection times. The results show that oral administration with ELP-DuIFN-α could provide better protection from co-infection or post-infection with DHAV-1 than pre-infection. This may be due to the cellular immune response caused by DHAV-1, which helps to enhance the effect of interferon.

The relative transcription levels of ISGs showed that except for Mx, the transcription levels of OAS, PKR, ZAP and ISG15 in co-infection or post-infection groups were higher than those in pre-infection group. This was consistent with the results of virus infection protection assays in ducklings. When compared with non-infected ducklings, all of the transcription levels of ISGs were significantly higher. This indicated that the ELP-fused DuIFN-α has in vivo immunological activity and has high potential to be developed as a therapeutic method.

These experimental data suggest that oral administration with ELP-DuIFN-α has the potential to control DHAV-1 infection. Alternatively, ELP-DuIFN-α can be orally administered during the interim of vaccination to enhance the protective immune response.

## Figures and Tables

**Table 1:** Primer sequences information.

Gene	Primer Sequence (5′-3′)
GAPDH	F:GCACTGTCAAGGCTGAGAACG R:GATGATAACACGCTTAGCACCAC
OAS	F:GCGGTGAAGCAGACGGTGAA R:CGATGATGGCGAGGATGTG
Mx	F:AACGCTGCTCAGGTCAGAAT R:GTGAAGCACATCCAAAAGCA
PKR	F:CCTCTGCTGGCCTTACTGTCA R:AAGAGAGGCAGAAGGAATAATTTGCC
ZAP	F:ATCGCTTTACCTTTCCTTG R:GTGCCATCGTATCATCTTCA
ISG15	F:TCGCAGCAGCTCCTATGAGGTC R:GCCAGAACTGGTCCGCTTGC

**Table 2:** Detection of cytotoxicity of ELP-DuIFNα.

ELP-DuIFNα (µg/mL)	Growth Inhibition (%)	
	MDCK Cells	DEF Cells
200	0	0
20	0	0
2	0	0
2 × 10^−1^	0	0
2 × 10^−2^	0	0
2 × 10^−3^	0	0
2 × 10^−4^	0	0
2 × 10^−5^	0	0
2 × 10^−6^	0	0
2 × 10^−7^	0	0

**Table 3:** In vitro antiviral activity of ELP-DuIFN-α on different cells.

Testing System	Antiviral Activity (IU/mg Protein)
MDCK-VSV	1.25 × 10^6^
DEF-VSV	1.25 × 10^7^
DEF-DHAV-1	6.0 × 10^4^

**Table 4:** Protection of duck embryos from DHAV-1 infection by ELP-DuIFN-α.

Group	No. of Duck Embryos	Death of Duck Embryos	Protection (%)
Normal control	5	0	
Challenge control	5	5	0
Treatment 1	5	0	100
Treatment 2	5	0	100
Treatment 3	5	1	80

**Table 5:** Protection of ducklings from DHAV-1 infection by ELP-DuIFN-α.

Group	No. of Ducklings	Route of Treatment	Time of Treatment	Death of Ducklings	Protection Rate (%)
Normal control	5			0	
Challenge control	5			5	
Treatment 1	5	Intramuscular	Co-infection	2	60
Treatment 2	5	Oral	Co-infection	1	80
Treatment 3	5	Oral	Pre-infection	2	60
Treatment 4	5	Oral	Co-infection	1	80
Treatment 5	5	Oral	Post-infection	1	80

## Data Availability

Not applicable.
